# A light-responsive multienzyme complex combining cascade enzymes within a peptide-based matrix†

**DOI:** 10.1039/c7ra10372g

**Published:** 2018-02-06

**Authors:** Yutong Wang, Yifei Zhang, Mengfan Wang, Yanan Zhao, Wei Qi, Rongxin Su, Zhimin He

**Affiliations:** State Key Laboratory of Chemical Engineering, School of Chemical Engineering and Technology, Tianjin University Tianjin 300072 P. R. China mwang@tju.edu.cn qiwei@tju.edu.cn; Tianjin Key Laboratory of Membrane Science and Desalination Technology Tianjin300072 P. R. China; The Co-Innovation Centre of Chemistry and Chemical Engineering of Tianjin Tianjin 300072 P. R. China

## Abstract

In this study, a light-responsive multienzyme complex (GOx&hemin@PepM) was developed by incorporating glucose oxidase (GOx) and hemin within a peptide-based matrix. An azobenzene group (Azo) was linked to the N-terminucs of glycine-phenylalanine-glycine tripeptide (GFG) to facilitate the formation of the supramolecular peptide-based matrix. Due to the proximity effect of GOx and hemin in the matrix as well as the biomimetic microenvironment of the peptide-based material, the transfer of intermediates can be enhanced and the catalytic activity of this multienzyme complex was greatly improved over free enzymes for catalyzing a cascade reaction. In addition, based on the light-responsive conformational switching of azobenzene between *E* and *Z* forms, the structure of the peptide-based matrix can be modulated, by which the catalytic activity of the multienzyme complex can be further controlled using UV and visible light. This study provides a new approach for constructing a stimuli-responsive multienzyme complex based on an adjustable material platform.

## Abstract 2

A light-responsive multienzyme complex was developed by incorporating glucose oxidase and hemin within a peptide-based matrix, in which the catalytic activity of multienzyme complex can be controlled using UV and visible light.

## Graphical abstract

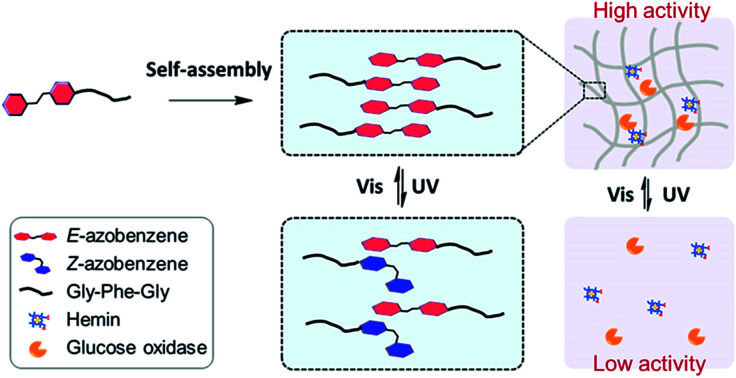

## Introduction

Cascade reactions are common in all living organisms; a series of chemical reactions is carried out in an order such that the product of one reaction is the substrate for another.^1–3^ Organelles can structurally confine enzymes in a proximal location, which facilitates the diffusion of intermediates among enzymes, thus enhancing the overall reaction efficiency.^4,5^ Inspired by *in vivo* cascade reactions, many efforts have been made to construct highly efficient multienzyme complexes by combining cascade enzymes within a confined matrix.^6^ Liu *et al.* developed a nanocomplex containing alcohol oxidase and catalase by directed assembly of enzymes with DNA and encapsulation with a polymer. Sun *et al.* developed a multienzyme catalyst by immobilizing an elastin-like polypeptide (ELP)-modified d-amino acid oxidase (DAAO) on hematin-functionalized multiwalled carbon nanotubes (MWCNTs), which exhibited higher catalytic efficiency than free ELP-DAAO.^7^

Recently, the self-assembly of short peptides has attracted growing attention in constructing micro- and nanostructural matrices due to its favorable stability, suitable variability and good biocompatibility.^8,9^ Peptide molecules can interact with each other through hydrophobic, π–π stacking, hydrogen bonding or ionic interactions, leading to the formation of various structures, such as nanofibrils, nanoparticles or nanotubes.^10,11^ In addition, the obtained supramolecular structure also provides a protein-like microenvironment that can facilitate biomimetic applications.^12–14^

**Scheme 1:**
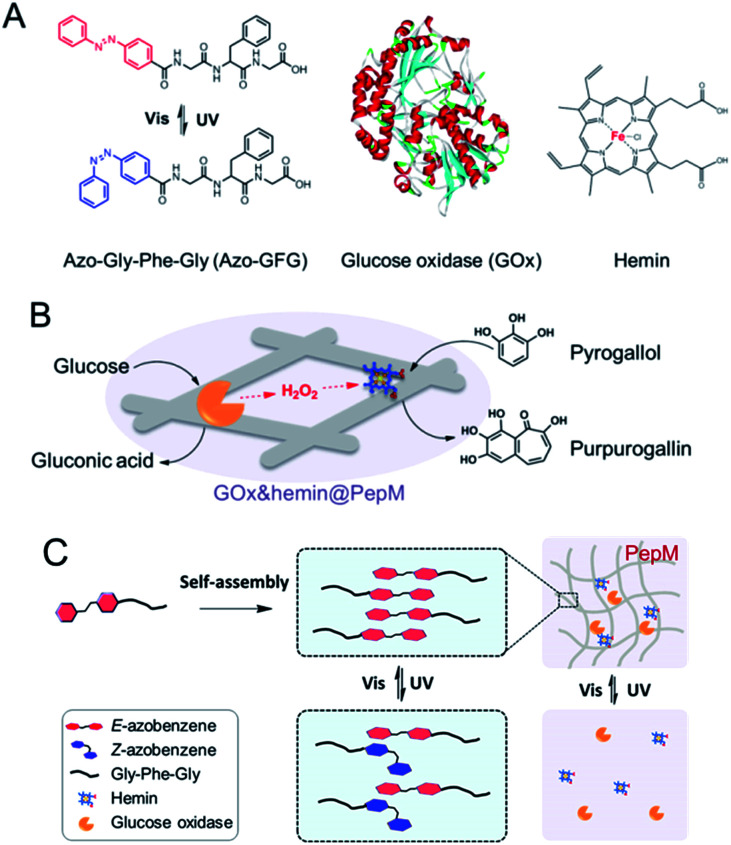
(A) Molecular structures of Azo-GFG, GOx and hemin. (B) Schematic illustration of the cascade reaction catalyzed by GOx&hemin@PepM. (C) Light-responsive property of GOx&hemin@PepM.

Cascade enzyme systems containing oxidases (such as glucose oxidase, urate oxidase and amino acid oxidase) and catalases are widely used in disease diagnoses, food processing and chemical synthesis.^15–18^ In this study, we created a novel light-responsive multienzyme complex (GOx&hemin@PepM) by incorporating glucose oxidase (GOx) and hemin within the azobenzene modified peptide-based matrix (PepM). Hemin is the prosthetic group of catalase, which is an iron-containing porphyrin and can be used individually as a molecular catalyst. The glycine-phenylalanine-glycine tripeptide (GFG) was designed with an aromatic azobenzene group (Azo) linked to the N-terminus to facilitate π–π stacking and the formation of the supramolecular matrix (Scheme 1A). The obtained peptide-based matrix provides a confined space for GOx and hemin. A cascade reaction using glucose and pyrogallol as the substrates was carried out to evaluate the catalytic activity of GOx&hemin@PepM. In the first reaction, glucose was oxidized by GOx and produced the intermediate H_2_O_2_. Then, H_2_O_2_ was consumed in the second reaction to convert pyrogallol into purpurogallin, with catalysis by hemin (Scheme 1B). Specifically, since the light-responsiveness of azobenzene group conformationally switches between the *E* and *Z* forms, the assembly and disassembly behavior of PepM as well as the consequent activity effect on GOx&hemin@PepM were also discussed (Scheme 1C). To our knowledge, this is the first time responsive peptide-based material was used to create a multienzyme complex with adjustable catalytic activity.

## Experimental section

### Materials

Azo-Gly-Phe-Gly (Azo-GFG) was purchased from Wuxi AppTec (Wuxi, China). Glucose oxidase (GOx, 100 U mg^−1^) and hemin (98%) were purchased from Aladdin Reagent Co. (Shanghai, China). All other chemicals were of analytical grade and obtained from commercial sources. Ultrapure water was used throughout the experiments.

### Preparation of PepM and GOx&hemin@PepM

Azo-GFG (2.44 mg, 5 μmol) was dissolved in 200 μL of methanol as the stock solution. For the preparation of PepM, 200 μL of stock solution was dropped into 1 mL of phosphate-buffered solution (PBS, pH 7.4). After vortexing for 2 min and incubating for 24 h at room temperature without disturbance, PepM was obtained.

For the preparation of GOx&hemin@PepM, a hemin solution was prepared by adding hemin (6.5 mg, 0.01 mmol) and sodium carbonate (10.6 mg, 0.1 mmol) into 1 mL of ultrapure water and heating at 60 °C until the solution became clear. Then, 80 μL of as-prepared fresh hemin solution, 30 μL of GOx solution (20 mg mL^−1^) and 200 μL of stock solution were added into 1 mL of PBS (pH 7.4) and vortexed for 2 min. The mixture was incubated at room temperature for 24 h without disturbance, leading to the formation of GOx&hemin@PepM.

## Characterization

The morphologies were characterized using an S-4800 field-emission scanning electron microscope (SEM; Hitachi High-Technologies, Japan) at an acceleration voltage of 3 kV. All samples were sputter-coated with platinum on an E1045 Pt-coater (Hitachi High-Technologies, Japan) before evaluation. The zeta potential values were measured on a Malvern Nano ZS (Malvern, England). Transmission electron microscopy (TEM; JEOL Ltd., Japan) images were recorded on JEOL 100CX-II at an acceleration voltage of 200 kV. Circular dichroism (CD) spectra were obtained on a J-810 CD spectropolarimeter (JASCO, Japan) with a 0.2 mm path length quartz cell at room temperature. The wavelength range was set to 190–500 nm, and the scan speed was 100 nm min^−1^. UV-vis spectra were obtained on a SpectraMAX 190 ultraviolet-visible spectrophotometer (Molecular Devices, US) with a 2 mm path length quartz cell and a wavelength range of 220–700 nm.

### Catalytic activity assay

The release rate of purpurogallin was used to represent the activity of GOx&hemin@PepM in the cascade reaction. In a typical experiment, 300 μL of GOx&hemin@PepM was added into 300 μL of a reaction solution containing 9 μmol of glucose and 4 μmol of pyrogallol in 50 mM PBS, pH 7.4. The reaction was maintained at 25 °C and the absorbance at 420 nm (OD_420 nm_) was monitored by a spectrometer (SpectraMax 190, Molecular Devices, US) to determine the release of purpurogallin. The activity was defined as the initial production rate of purpurogallin (μM min^−1^) catalyzed by GOx&hemin@PepM.

### Kinetic analysis

Steady state assays were carried out at 25 °C when 150 μL of GOx&hemin@PepM was added into 650 μL reaction solution (50 mM PBS, pH 7.4) containing different concentration of substrates. To investigate the kinetic mechanism of the multiple substrate cascade reaction, the assays were performed with a fixed concentration of one substrate and varying the concentration of the other. As comparison, the kinetic analysis of free enzymes was also carried out with the same amount of free GOx and free hemin to that in GOx&hemin@PepM.

## Results and discussion

### The formation of PepM through the self-assembly of Azo-GFG

The importance of aromatic stacking has been illustrated for many peptide assembly systems, where the aromatic group that linked to the N-terminus (*e.g.*, Fmoc, naphthalene or pyrene) is a consistent facilitator for peptide self-assembly.^19,20^ Herein, a functional aromatic group, azobenzene, was attached to the N-terminus with glycine (G) as a flexible linker. Phenylalanine (F) is a commonly used amino acid in peptide assembly materials because its aromatic side-chain can also form inter-molecular π–π stacking. The glycine at the C-terminus provides a –COOH tail to enhance the hydrophilicity of the peptide and facilitate the formation of hydrogen bonds.

**Fig. 1:**
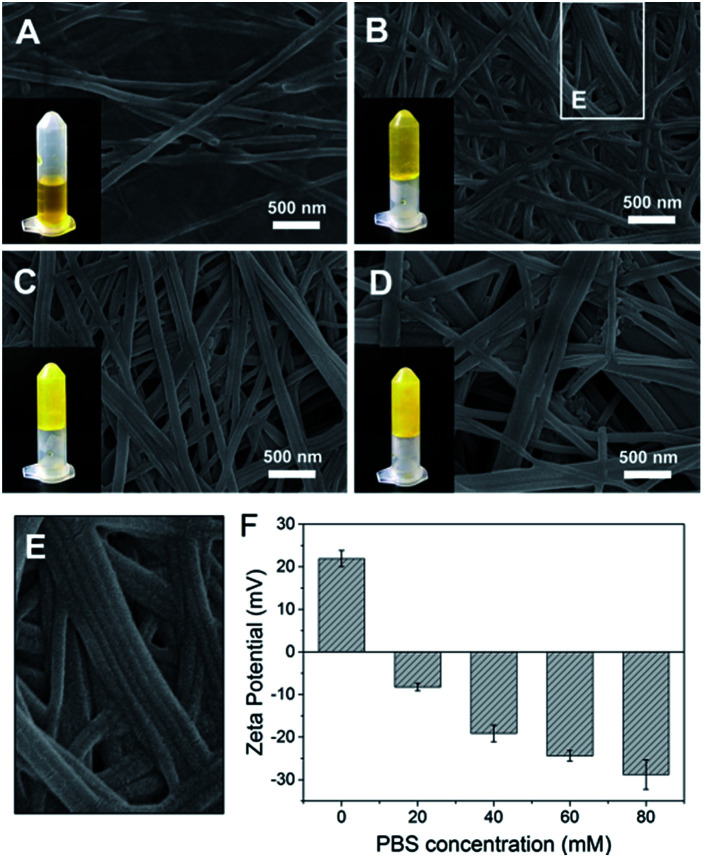
SEM images of Azo-GFG hydrogel formed in PBS at salt concentrations of 20 mM (A), 40 mM (B), 60 mM (C) and 80 mM (D). Magnified area in (B) (E). Zeta potential analysis of the Azo-GFG hydrogel formed in water and PBS (F).

Azo-GFG was found to be soluble in water, but it formed a yellow hydrogel in PBS, indicating that the self-assembly of Azo-GFG is salt dependent. To eliminate the interference of Na_2_HPO_4_/NaH_2_PO_4_ on pH, different concentrations of PBS with the same pH (pH 7.4) were used. As shown in Fig. 1, the concentration of Na_2_HPO_4_/NaH_2_PO_4_ affects the formation of the hydrogel. When the peptide stock solution was dropped into 20 mM PBS (Fig. 1A), the Azo-GFG solution remained soluble and could not form a hydrogel, even after 48 h. SEM images reveal that only a few nanofibers appeared with diameters of 150 nm, which was too loose to form a stable fibrous network. When the PBS concentration was increased to 40 mM, more nanofibers were generated (Fig. 1B). Some nanofibers began to parallel align to form nanoribbons, which can be observed in the bordered magnified area in Fig. 1E.^21^ These flexible nanofibers and nanoribbons can easily overlap and entangle with each other to form networks. Thus, a hydrogel was obtained at this PBS concentration. By increasing PBS to 60–80 mM, the aggregation of adjacent nanofibers increased greatly (Fig. 1C and D), forming bundles of nanoribbons. The diameter of the nanoribbons further increased to 200–400 nm, which promoted the formation of a stable hydrogel. Zeta potential was detected to investigate the effect of PBS on the form of nanofiber, as shown in Fig. 1F. In pure water, the peptide was positively charged (+21.9 mV) which might due to the protons that attracted by the lone pair electron on nitrogen in the azobenzene group, and thus resisted the formation of supramolecular fibers. The addition of high ionic strength buffers or polyvalent anions can specifically relieve molecular resistance, shield positive or negative charges and allow the formation of long-range microscopic fibers.^22,23^ Due to multivalent properties, PO_4_^3−^ interacts with the positively charged Azo-GFG, reducing the charge–charge repulsion and promoting intra- and inter-peptide cross-linking. Increasing the PO_4_^3−^ buffer ionic strength from 20 to 80 mM resulted in a significant decrease in zeta potential, demonstrating the accumulation of PO_4_^3−^ on nanofibers during the self-assembly. As a result, 50 mM PBS was adopted to fabricate the peptide-based matrix due to its proper interlaced network structure for hosting catalysts.

### Characterization and catalytic activity of GOx&hemin@PepM

**Fig. 2:**
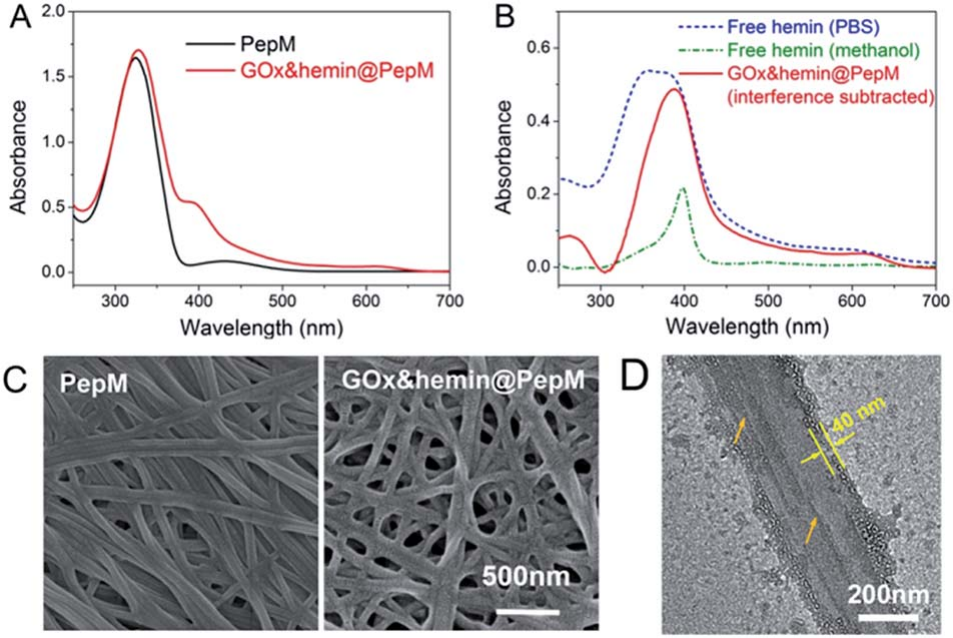
(A) UV-vis spectrum of PepM and GOx&hemin@PepM. (B) UV-vis spectrum of hemin in PBS, methanol and PepM. (C) SEM images of PepM and GOx&hemin@PepM. (D) TEM image of the GOx&hemin@PepM.

Based on this proteic network structure, a biocompatible protocol was adopted to prepare GOx&hemin@PepM by incorporating GOx and hemin within PepM. Fig. 2A demonstrates the UV-vis spectrum of pure PepM and GOx&hemin@PepM in PBS buffer (pH 7.4). For PepM, the characteristic peaks approximately 330 nm and 420 nm were ascribed to the π–π* absorption band of *E*-azobenzene and *Z*-azobenzene, respectively, which demonstrated the dominant *E*-configuration in PepM.^24,25^ When PepM was used to host GOx and hemin (GOx&hemin@PepM), the strong absorption at 330 nm was retained, indicating that the incorporation of GOx and hemin did not affect the configuration of azobenzene. The shoulder at approximately 400 nm appearing in GOx&hemin@PepM comes from the hemin molecules. The direct application of hemin in aqueous solution is a significant challenge because hemin will aggregate into catalytically inactive dimers and self-destruct in oxidizing media.^26^ However, the hemin that dissolves in organic reagents, such as methanol or dimethyl sulfoxide, or that exists in natural catalase maintains its active monomeric form. To investigate the molecular form of hemin in GOx&hemin@PepM, the interferential spectrum of PepM was subtracted from that of GOx&hemin@PepM (Fig. 2B). The free hemin in PBS demonstrated a broad Soret peak at 346–400 nm, suggesting a mixture of inactive dimeric hemin and active monomeric hemin.^12^ Unlike in PBS, the methanol-dissolved hemin displays a single Soret band at 400 nm, which corresponds to monomeric hemin. Compared with free hemin in PBS and methanol, the spectrum of hemin in PepM is similar to that in methanol, indicating that most of hemin is in the monomeric form in GOx&hemin@PepM. The role of PepM that preserves the hemin as monomeric form can also be confirmed through titrating the hemin with different concentrations of PepM. Fig. S1 in ESI† revealed that with the adding of PepM, the Soret peak at 346 nm dropped obviously, whereas the peak at 400 nm grew, indicating the transfer of hemin from dimeric to monomeric form. This role might come from the proteic nature of peptide-based matrix, which effectively inhibits the aggregation of hemin through supramolecular interactions, similar to in a natural catalase molecule.

Scanning electron microscope (SEM) was used to investigate the morphology of GOx&hemin@PepM. As shown in Fig. 2C, the incorporation of catalysts did not affect the intrinsic network structure of the peptide-based matrix, but the surface of the nanofibers became rougher. We also observed the increased porosity of GOx&hemin@PepM than PepM. This might due to the loaded GOx and hemin that reduced the superficial affinity among nanofibers and suppressed the close entanglement of nanofibers. As a result, the pores facilitated the mass transfer between reactants and catalysts. Transmission electron microscopy (TEM) was further used to analyse the nanofibers in GOx&hemin@PepM (Fig. 2D). The nanofiber is approximately 300 nm in width and composed of several thin parallel nanofibers. The orange arrows indicated the boundary of the thin fibers. As a comparison, Fig S2† showed a single strand nanofiber which did not display any laminated structure. In addition, the nanofibers were covered by a thin layer, approximately 40 nm thick. This layer might contribute to the rough surface of nanofibers and was speculated the place where GOx and hemin located. The energy dispersive X-ray spectrometry (EDX) analysis confirmed the presence of sulfur and iron on the nanofibers which indicated that the GOx and hemin were steadily attached to the nanofibers in GOx&hemin@PepM (Fig. S3†).

To evaluate the catalytic activity of GOx&hemin@PepM, a cascade reaction was performed under mild conditions. In the reaction (I), GOx catalyzed the oxidation of glucose, producing the intermediate product H_2_O_2_. Then, H_2_O_2_ was transferred to hemin and began the second oxidation reaction toward pyrogallol to produce chromogenic purpurogallin:I
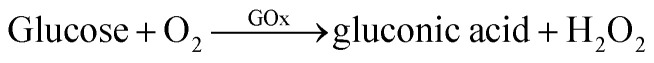
II
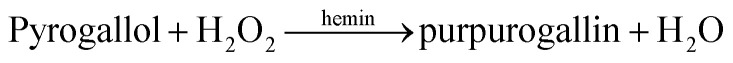


**Fig. 3:**
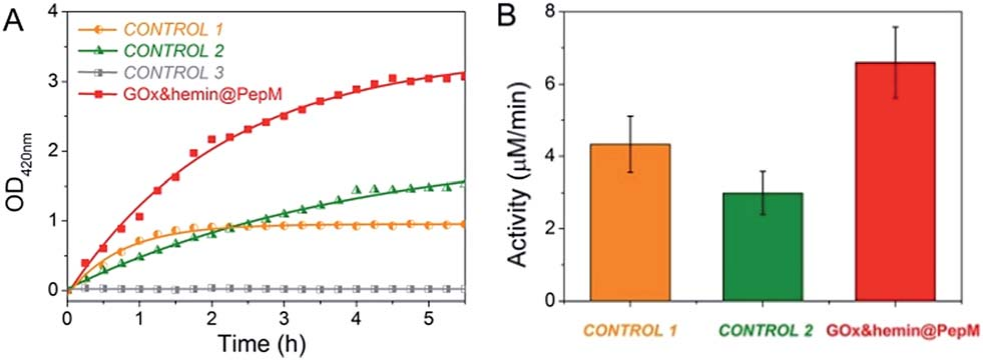
(A) Plots of absorbance *vs.* time for the cascade reaction catalyzed by GOx&hemin@PepM, CONTROL 1, CONTROL 2 and CONTROL 3. For CONTROL 1, GOx and hemin were simply dissolved in substrate solution. For CONTROL 2, GOx was dissolved in substrate solution and hemin was incorporated in PepM. For CONTROL 3, pure PepM was added without GOx or hemin. (B) Catalytic activity of GOx&hemin@PepM, CONTROL 1 and CONTROL 2.

The increased absorbance at 420 nm can be monitored over time to evaluate the catalytic activity of GOx&hemin@PepM in the cascade reaction. As controls, the same amount of GOx and hemin in the form of free GOx + free hemin (CONTROL 1), free GOx + hemin@PepM (CONTROL 2) and pure PepM (CONTROL 3) were also studied, as shown in Fig. 3. When GOx and hemin were both simply mixed with free substrates (CONTROL 1), the OD_420 nm_ value increased rapidly in the first 1.5 h but remained constant afterwards. This indicates that although the catalysts in CONTROL 1 displayed favorable activity (4.33 μM min^−1^) in the first 1.5 h, they were easily inactivated in the long-time reaction. As discussed above, free hemin is in a low-activity dimer form when dissolved in aqueous solution, and it can be easily disassembled in the oxidizing media.^26^ In the solution environment of CONTROL 1, GOx and hemin are distributed freely in the medium, and there was no spatial confinement of catalysts to provide a close distance for H_2_O_2_ transfer. Therefore, large amount of H_2_O_2_ was released from GOx and accumulated in the reaction medium, further inhibiting the activity of hemin. In the case of CONTROL 2, hemin was incorporated into PepM (hemin@PepM), which ensures the catalytic activity of hemin. However, there is also a serious mass-transfer limitation for H_2_O_2_ to move from the bulk solution into the matrix. Therefore, although the catalysts were active throughout the whole reaction, the activity was still in a low level (2.99 μM min^−1^). For CONTROL 3, pure PepM was mixed with substrates and the unchanged absorbance indicated no instinct catalytic ability of peptide matrix. Compared with the controls, GOx&hemin@PepM exhibited excellent catalytic performance. The OD_420 nm_ value increased rapidly during the whole reaction, implying the H_2_O_2_ could be efficiently transferred and consumed within GOx&hemin@PepM. This comes from the synergism of the proximity effect and the highly active hemin, which were provided by the peptide-based matrix. As a result, the catalytic activity of GOx&hemin@PepM is 6.67 μM min^−1^.

**Fig. 4:**
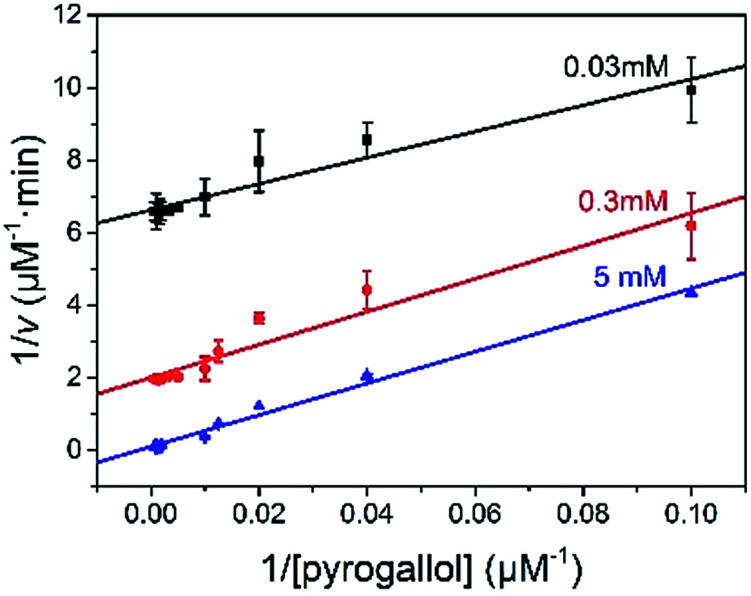
Lineweaver–Burk plots for the cascade reaction catalysed by GOx&hemin@PepM. The glucose concentration was 0.03, 0.3 and 5 mM, respectively.

The reaction kinetic for the two substrates cascade reaction catalysed by GOx&hemin@PepM was investigated. The typical Michaelis–Menten curves were found to each substrate over a large concentration range (Fig. S4†). The linear Lineweaver–Burk curves in Fig. 4 implied the enzyme-like characteristic of hemin in GOx&hemin@PepM, which might owe to the proteic microenvironment in peptide-based matrix. Moreover, the parallel double reciprocal plots under different glucose concentrations indicated that the multienzyme complex followed the Ping-Pong multiple substrates catalysis mechanism. Table 1 compared the kinetic parameters of GOx&hemin@PepM with free GOx + free hemin. It can be seen that when at the same glucose concentration, GOx&hemin@PepM exhibited lower *K*_m_ value than free enzymes, implying the stronger affinity towards pyrogallol. When at the same pyrogallol concentration, GOx&hemin@PepM showed higher *V*_max_ value, indicating the higher conversion efficiency of multienzyme complex.

**Table 1:** The kinetic parameters of GOx&hemin@PepM and free enzymes

	Substrate	*K*_m_ (mM)	*V*_max_ (μM min^−1^)
GOx&hemin@PepM	5 mM glucose	0.43 ± 0.029	9.90 ± 1.09
GOx&hemin@PepM	6.8 mM pyrogallol	0.55 ± 0.032	17.05 ± 0.98
Free GOx + hemin	5 mM glucose	1.17 ± 0.029	9.42 ± 2.18
Free GOx + hemin	6.8 mM pyrogallol	0.57 ± 0.033	8.86 ± 0.22

### Light-responsiveness of GOx&hemin@PepM

**Fig. 5:**
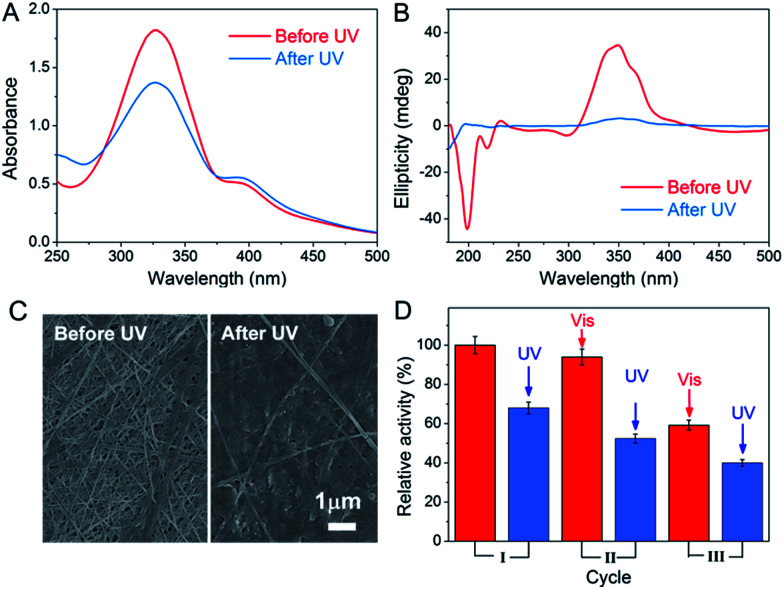
UV-vis spectra (A), CD spectra (B) and SEM images (C) of GOx&hemin@PepM before and after UV irradiation. (D) Activity of GOx&hemin@PepM after repeated UV and visible light irradiation.

The light-responsive property of azobenzene was exploited due to the *E*/*Z* conformational switches that can be modulated by UV and visible light. In this study, light was also found to affect the formation of the peptide-based matrix and the catalytic activity of GOx&hemin@PepM. When GOx&hemin@PepM was exposed to UV light, the hydrogel began to disintegrate and completely turned into solution within 12 h. The UV-vis spectrum was used to investigate the conformational change of Azo-GFG during the gel–sol transition process. It can be seen from Fig. 5A that after UV irradiation, GOx&hemin@PepM displayed a distinct drop in the adsorption at approximately 330 nm, along with an increase at 260 nm and 430 nm, which were ascribed to the π–π* and n–π* bands of *Z*-azobenzene, respectively. This confirmed the conformational switch of the azobenzene moiety in Azo-GFG from the *E* to the *Z*-form. The secondary structure of the Azo-GFG backbone was analyzed by CD, as shown in Fig. 5B. The extinction of the broad signal approximately 360 nm confirms the reduction of π–π* staking of *E*-azobenzene. Additionally, for GOx&hemin@PepM before UV irradiation, the strong negative peaks at 205 nm and 222 nm were assigned to the α-helix structure of the peptide backbone. After UV irradiation, the characteristic structure of the α-helix faded. Since the primary driving force of the Azo-GFG assembly is π–π stacking among *E*-azobenzene groups, the configuration change of azobenzene directly affects the molecule arrangement in GOx&hemin@PepM, destroying the supramolecular α-helical structure and resulting in the disassembly of the peptide-based matrix. Fig. 5C demonstrates the morphological change of GOx&hemin@PepM before and after UV irradiation. The original dense fibrous network disappeared and only a few nanofibers remained, which were too loose to load GOx and hemin.

As mentioned above, the high catalytic activity of GOx&hemin@PepM depends largely on the supramolecular structure of PepM to suppress dimeric hemin and enable hemin and GOx to approach each other and transfer H_2_O_2_ efficiently. Given that UV irritation destroys the supramolecular structure of PepM, GOx and hemin will be released from the matrix and act as free catalysts, similar to in CONTROL 1. As a result, the catalytic activity of GOx&hemin@PepM was clearly decreased to 4.48 μM min^−1^ after UV irradiation. Due to the reversibility of *E*- and *Z*-azobenzene under UV and visible irradiation, the catalytic activity under repeated UV-visible irradiation was also investigated. For each cycle, GOx&hemin@PepM was processed with 12 h UV irradiation to turn into solution, followed by a 24 h visible irradiation for reassembly into a hydrogel (see ESI, Fig. S5†). From Fig. 5D, the catalytic activity of the disassembled GOx&hemin@PepM can be recovered to some extent if exposed to visible light. Under visible light, *Z*-azobenzene slowly turns back into the *E* form, driving the free Azo-GFG to reassemble into the fibrous matrix and, at the same time, reincorporates GOx and hemin. However, we also observed that the reversible activity declined during repeated UV-visible light switches. In the third cycle, the reversible activity of GOx&hemin@PepM already dropped to 3.96 μM min^−1^, which was only 60% that of the initial value. This might be caused by the increasingly fragile structure of PepM and the deactivation of GOx and hemin during repeated irradiation processes.

## Conclusions

In summary, a multienzyme complex was developed by incorporating GOx and hemin within a peptide-based matrix. Upon the self-assembly of Azo-GFG, the peptide-based matrix displayed a fibrous network structure, which provides a confined space for the efficient transfer of intermediates between two catalysts and provides a biomimetic environment for hemin to retain high activity. Compared with the cascade reaction catalyzed by free GOx and hemin, the obtained GOx&hemin@PepM exhibits excellent activity during the whole reaction. In addition, based on the light-responsive property of azobenzene, the activity of GOx&hemin@PepM can be modulated by UV and visible light. This study provides a new strategy for constructing a multienzyme complex and also opens a novel approach for developing an adjustable material platform for enzyme activity control.

## Conflicts of interest

There are no conflicts to declare.

## Supplementary Material

RA-008-C7RA10372G-s001
