# Supplementary material for: A light-responsive multienzyme complex combining cascade enzymes within a peptide-based matrix
Source: RSC Adv. 2018 Feb 6;8(11):6047–52. doi: 10.1039/c7ra10372g (PMC9078227; doi:10.1039/c7ra10372g)
Supplement: RA-008-C7RA10372G-s001 [file RA-008-C7RA10372G-s001.pdf]

Electronic Supplementary Material (ESI) for RSC Advances.
This journal is © The Royal Society of Chemistry 2018

Supporting Materials

A light-responsive multienzyme complex by combining cascade enzymes within a peptide-based matrix

Yutong Wang,‡[a] Yifei Zhang,‡[a] Mengfan Wang,*[ab] Yanan Zhao,[a] Wei Qi,*[abc] Rongxin Su[abc] and Zhimin He[a]

[a.] State Key Laboratory of Chemical Engineering, School of Chemical Engineering and Technology, Tianjin University, Tianjin 300072, P. R. China.
E-mail: mwang@tju.edu.cn; qiwei@tju.edu.cn.

[b.] Tianjin Key Laboratory of Membrane Science and Desalination Technology, Tianjin300072, P. R. China.

[c.] The Co-Innovation Centre of Chemistry and Chemical Engineering of Tianjin, Tianjin 300072, P. R. China.

‡ These authors contributed equally to this work.

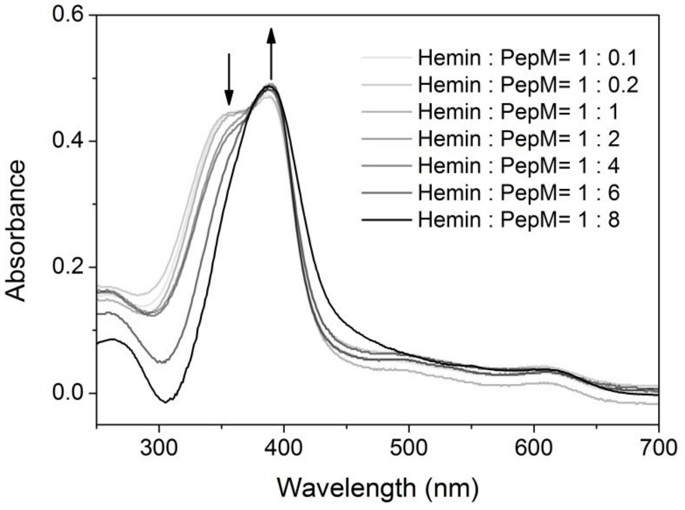

Fig S1 Titration of hemin with different concentrations of PepM.

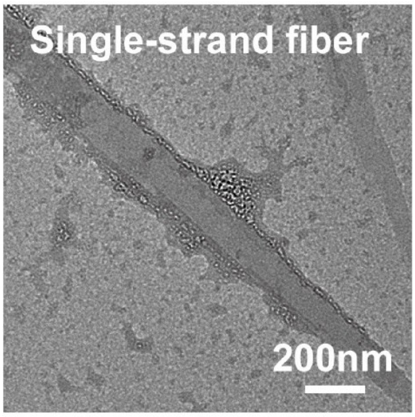

Fig S2 The morphology of single-strand nanofiber in GOx&hemin@PepM.

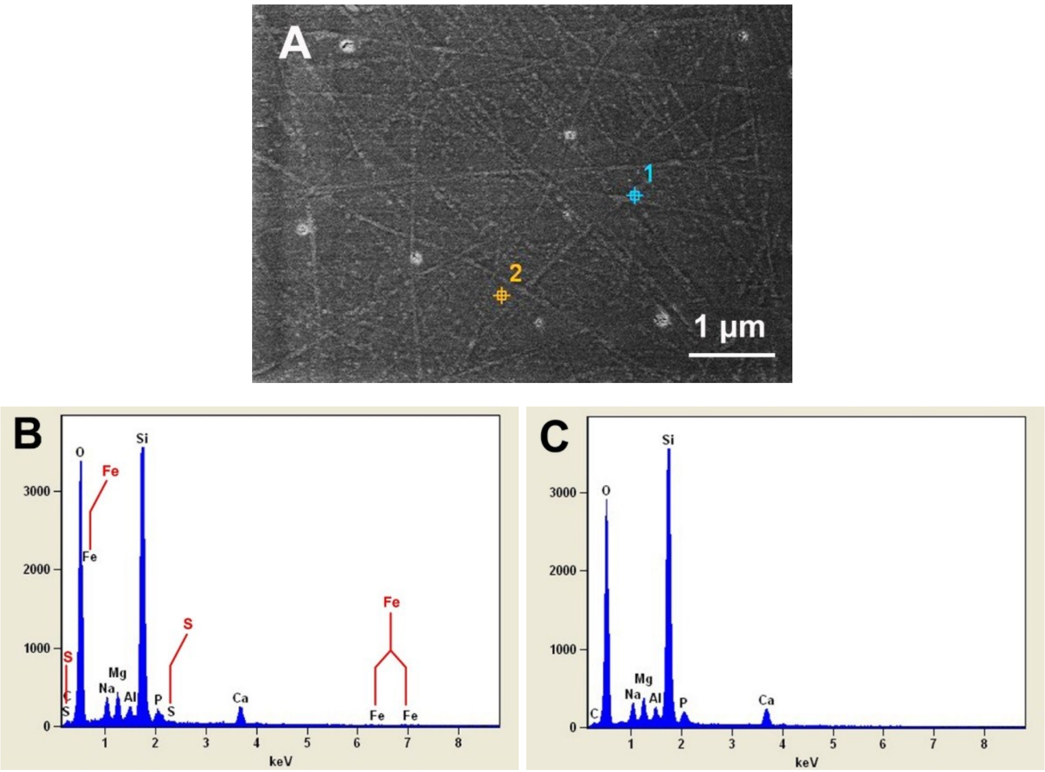

Fig. S3 (A) Scanning area of EDX analysis. *POINT 1* is the crossing area of the GOx&hemin@PepM nanofibers. *POINT 2* is the blank space exposing the glass substrate slide. (B) EDX analysis of *POINT 1*. (C) EDX analysis of *POINT 2*.

As shown in Fig. S1, point scanning EDX analysis was used on the cross point of nanofibers (*POINT 1*) and blank area (*POINT 2*). Both areas showed high peaks at ~0.4 keV, ~1.8 keV and ~3.7 keV, indicating the presence of O, Si and Ca respectively, which are rich in glass substrate slides. Compared with *POINT 2*, the characteristic Fe and S peaks can be detected at *POINT 1*, confirming the residence of GOx and hemin in this area.

For iron, there are three peaks at ~0.5 keV and 6-7keV, corresponding to the energy excitation from different electronic shells of iron atoms. The resulting element Wt.% of iron and sulfur are approximately 0.04% and 0.06%, approximately in agreement with the initial concentration of these two elements.

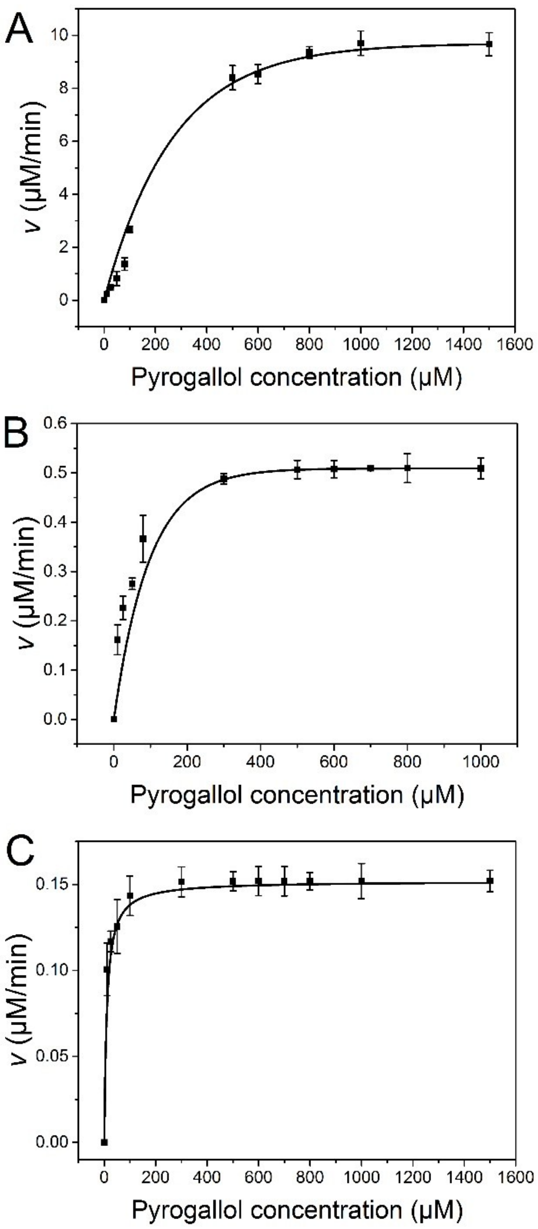

Fig. S4 Kinetic analysis of GOx&hemin@PepM. Reaction was measured at room temperature using 150 μL of multienzyme complex in 800 μL PBS (50mM, pH7.4) with the fixed glucose concentration of (A) 5 mM, (B) 0.3 mM and (C) 0.03 mM.

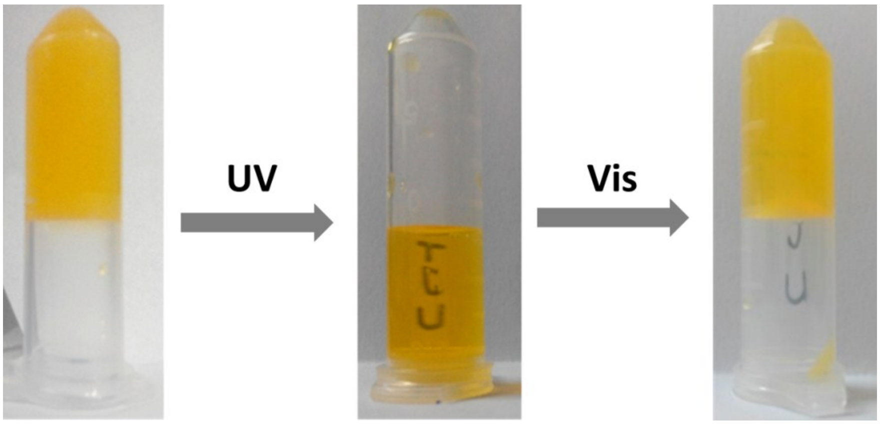

Fig. S5 Hydrogel-solution transformation of GOx&hemin@PepM under UV and visible light irradiation.
